# 

**Published:** 1884-06

**Authors:** 


### INDEX TO VOLUME XVIII.

PAGE.

A.

Actinomycosis, Discovered in American Cattle, W. T. Belfield ... 165
Adonis Vernalis, Properties of ... 317
Aetiology of Congenital Deformities of the Bulbs of the Eye ... 99
Alcoholic Paralysis and Cerebral Syphilis ... 249
Alteration of the Inferior Vena Cava ... 309
Anæmia Acute, Successful Injection of a Solution of Salt, 110
Aneurysma Traumaticum ... 296
Antisepsis from a Later Standpoint ... 443
Atropine, a Rare Case of Poisoning by ... 73

B

Bartholow, Roberts, Review ... 666
Becker, W. T., Interesting case of Repair of Tissue after Injury ... 37
Belfield, W. T., Actinomycosis in American Cattle ... 165
Bennett, E. R., Letter ... 664
Beriberi ... 310
Blackburn, G. E., Letter ... 665
Bloody Tears ... 301
Books and Pamphlets Rec'd ... 90, 446
British Medical Association—The next meeting ... 278
Brown, J., Selection ... 671
Byford, W. H., Doctorate Address ... 561

PAGE.

C.

Canadian Medical Degrees in England ... 193
Case of Monstrosity, Hard ... 246
Case of Recovery after Extensive Contusion and Laceration of the Perinæum and Urethra, Washburne ... 468
Case of Resection of the Elbow Joint, W. H. Washburne ... 131
Case of Retention of Placenta Succenturiata, Haussmann ... 245
Cerebral Syphilis ... 318
Chicago Medical College Commencement ... 556
Chicago Medical Society:
- Meeting Nov. 5, 1883 ... 167
- Meeting Nov. 19, 1883 ... 74
- Meeting Dec. 17, 1883 ... 172
- Meeting Jan. 7, 1884 ... 263
- Meeting Jan. 14, 1884 ... 420
- Meeting Jan. 21, 1884 ... 267
- Meeting Feb. 4, 1884 ... 420
- Meeting Feb. 18, 1884 ... 427
- Meeting Mar. 3, 1884 ... 510
- Meeting Mar. 17, 1884 ... 631
- Meeting April 7, 1884 ... 643

Chicago Pathological Society:
- Meeting Oct. 8, 1883 ... 410
- Meeting Nov. 10, 1883 ... 412
- Meeting Dec. 10, 1883 ... 415

Chicago Society of Ophthalmology and Otology:
- Meeting Feb. 14, 1884 ... 505

Chinese Method of Determining Paternity ... 517
Chloride of Gold ... 186
Circulation in the Cornea ... 302
Clevenger, S. V., Our Insane ... 139
Clevenger, S. V., Selection ... 324

PAGE.

Clevenger, S. V., Clinical and Pathological Reports of Cases of Insanity, 113, 230, 384, 474, 583
Coated Tongues 333
Coffee in Strangulated Hernia 81
Colburn, J. Elliot, Treatment of Trichiasis by Electrolysis 39
Collective Investigation of Disease 82
Complete Handbook of Treatment, Review 287
Concerted Action by State Boards of Health 166
Constipation, Treatment of by other than Medicinal Measures, Henry N. Josselyn 41
Construction of Hospitals for the Insane, J. C. Spray 390
Cook Co. Hospital Report, 71, 248
Correspondence 92
Correspondence, Domestic, 184, 282, 434, 518, 661
Correspondence, Foreign 180
Correspondence, Special 521
Count Rumford 87
Creosote, Treatment of Dental Caries with 186
Criticising the Journal of the American Medical Association 195
Crothers, T. D., Letter 285
Currie, C. E., Hospital Report 71
Cysticercus in the Vitreous Humor 308

D.

Davis, E. P., Hospital Report 248
Davis, C. G., Gelsemium Sempervirens 610
Dearborn, Dr., Editorial 658
Decalcified Bone Drainage Tubes 333
Decapitation, New Observations on 291
Decision upon an Anatomy Act 433
Dental Journal Association 286
Dentigerous Cyst of the Inferior Maxilla, John S. Marshall 28
Disadvantages of the Upright Position, Clevenger 324

PAGE.

Differential Diagnosis of Lupus, Lepra and Cancer in the Throat 100
Diphtheria on Pregnancy 305
Diphtheria, Prolonged 249
Diphtheria, Treatment of with the Cyanide of Mercury 102
Doctorate Address, Byford 561
Do the Contractions of the Uterus depend upon the Cerebro-Spinal System 313

E.

Echinococcus Alveolaris of the Liver 314
Eczema and its Management, Review 526
Eczema, Treatment of, Reynolds 240
Editorials, 82, 187, 278, 443 658
Elder, Thomas A., Letter 282
Electro Therapeutics, Review 667
Embolism of the Axillary Artery, A. H. Foster 400
Emetics or Brisk Cathartics in Strumous Ophthalmia. R. F. Henry 128
Encephalitis Congenita 298
Erb, Wilhelm, Review 667
Extraction of a Large Foreign Body, Situated in the Rectum, A. Lagorio 96

F.

Fitch, W. H., Abdominal Abcess 225
Fonda, D. B., Letter 93, 434
Foreign Body in the Uterus 316
Foster, Addison H., Embolism of the Axillary Artery 400
Four Million Pigs with Bad Grammar and Worse Logic, but No Disease 279
Fracture of the Cranium in a Child Four Months Old 293

G.

Gall Stone in Relation to Cancer of the Gall Bladder 295
Gangrene of the Bladder 293
Gelsemium Sempervirens, Davis 610

PAGE.

Glycerine in Fevers............. 303
Grönvold, Chr., Leprosy in Minnesota........................ 133
Gulstonian Lectures on Neuroses of the Viscera............ 538
Gunn, Moses, Reduction of Hip and Shoulder Dislocations................................ 449

H.

Hagenbach, A. W., Letter...... 661
Hagenbach, A. W., Practice at the County Infirmary......... 1
Hand-book of the Diagnosis and Treatment of Diseases of the Throat and Nose, Review................................ 289
Hard, C., Case of Monstrosity, 246
Haussmann, Dr., a Case of Retention of Placenta Succenturiata.............................. 245
Heart, Motor Derangement of, Service of T. Bidwell......... 71
Henry, G. R., Letter.............. 438
Henry, R. F., Emetics or Brisk Cathartics in Strumous Ophthalmia............................ 128
Hoang-Nan and its Therapeutical Indications................ 307
Hospital Reports..............71, 248
Hospital with Circular Wards, 671
Hotz, F. C., Experience with Jequirity in the Treatment of Granular Eyelids......... 121
Hunter, C. T., Obituary......... 651
Hutchinson, Jonathan, Note, 281
Hydrophobia, Virus of........... 522
Hymen, The, McKee............. 486
Hysteria Cured by Oöphorectomy................................ 300
Hysteria, Therapeutic Ataxia of.................................... 102

I.

Illinois State Board of Health, Meeting Jan. 17 and 18, 1884, 250
Illinois State Medical Society, 558
Ingals, E. Fletcher, Naso-Pharyngeal Fibromata........ 604
Insanity and its Treatment, Review................................ 439
Insanity, Review.................. 439
Interesting Case of Repair of Tissue after Injury, W. T. Becker.............................. 37

PAGE.

International Encyclopedia of Surgery, Review................ 527
Intoxication by Naphthaline, 294
Irregular or Hour-glass Contraction of the Uterus, McKee.............................. 365
Items...... ............................ 332

J.

Jaggard, W. W., Adherent Placenta ................................ 630
Jenks, E. W., Selection......... 530
Jequirity Ophthalmia............ 320
Jequirity, Experience with... 121
Josselyn, Henry N., On the Treatment of Habitual Constipation by other than Medicinal Measures............... 41

K.

Kiernan, Jas. G., Letter......... 663
Kiernan, Jas. G., Paretic Dementia................................ 574
Kiernan, Jas. G., Review........ 439
King, H. Kane, Correction...... 420
King, W. H. Kane, Report of a Case of Ovariotomy......... 35
Koch's Official Report of the Cholera in Egypt................ 213

L.

Lagorio, A., Extraction of a Large Foreign Body, Situated in the Rectum........... 96
Laryngitis Hypoglottica Hypertrophica......................... 319
Leprosy in Minnesota, Chr. Grönvold............................ 133
Lydston, G. Frank, Modern Sanitation.......................... 337
Lydston, G. Frank, Necessity for an Ambulance System in Chicago............................. 402

M.

Malaria, Prophylaxis of......... 95
Manaca, Note on.................. 247
Manual of Chemistry, Review 669
Marshall, John S., Dentegerous Cyst, Removal of............... 28
Materia Medica and Therapeutics, Review................. 666

PAGE.
McKee, E. S., Original Translation........ 199
McKee, E. S., Irregular or Hour-glass Contraction of the Uterus........ 365
McKee, E. S., The Hymen...... 486
Medical Jurisprudence, Review........ 439
Meeting of the International Medical Congress........ 279
Mexican Letter........ 180
Michigan State Board of Health, Meeting Jan. 8, '84.. 261
Michigan State Board of Health........ 655
Miliary Tuberculosis of the Spleen........ 304
Miscellaneous, 311, 334, 446, 556
Modern Sanitation, G. Frank Lydston........ 337
Morgan, Wm. E., Letter........ 664
Municipal Hygiene........ 219
My Experience with Jequirity in the Treatment of Granular Eyelids, Hotz........ 121
Myxosarcoma of the Pleura... 98

N.

Napthaline, Intoxication by... 294
Naso-Pharyngeal Fibromata, Ingals........ 604
Necessity for an Ambulance System in Chicago, Lydston 402
Nephrectomy........ 302
New Mode of Operating for Fistula in Ano........ 530
New York Neurological Society Meeting, March 4, 1884... 497
Northwestern Medical College 282

O.

Obituary, ........111, 657
Œsophagotomy........ 314
Official List Changes in Marine Hospital Service.....334, 560
Official List of Medical Officers Marine Hospital Service........ 336
On the Treatment of Habitual Constipation by other than Medicina Measures, Henry N. Josselyn........ 41
Oöphorectomy, Hysteria Cured by........ 300

PAGE.
Ophthalmia Jèquiritica, Pathology of........ 320
Original Communications, 1, 113, 225, 337, 449, 561
Original Translations, 96, 199, 291
Ovariotomy, Report of a Case of, W. H. Kane King........ 35
Our Insane, Clevenger........ 139

P.

Paretic Dementia and Life Insurance, Kiernan........ 574
Parker, Dr. Willard, Health of 290
Pathology and Treatment of Gonorrhœa, Review........ 524
Pathology, Diagnosis and Treatment of Diseases of Women, Review........ 197
Philosophy of and Manipulation in the Reduction of Hip and Shoulder Dislocations, Moses Gunn........ 449
Phthisis, Treatment of........ 299
Poisoning by Chloride of Potassium........ 304
Polyuria, Treatment of........ 110
Pott's Disease, C. E. Webster.. 357
Practical Manual of the Diseases of Children........ 289
Practical Treatment of Impotence, Sterility and allied Disorders, Review........ 288
Practice at the County Infirmary, A. W. Hagenbach........ 1
Pregnancy of 56 years........ 208
Prevention of Horse Accidents........ 179
Priapismus caused by Leukemia........ 81
Prolonged Diphtheria........ 249
Prophylaxis of Malaria........ 95
Psychological Medicine, Review........ 439

Q.

Quebracho and its Alkaloids... 97
Quiz Compends, Review........ 198

R.

Rachitic Thorax with Perverse Respiration........ 295
Ramsay, A. C. L., Washing out of the Stomach........ 32

PAGE.

Reflex Dilatation of the Pupils. 315
Regulating the Practice of Medicine in Colorado......... 281
Remarks on the Washing out of the Stomach, A. C. L. Ramsay.......................... 32
Removal of Naso-Pharyngeal Fibromata, Ingals.............. 604
Report of case of Ovariotomy, W. H. Kane King........ 35
Report of case of Malignant Stricture of the Descending Colon, Skeer.................... 408
Report of two cases of Abdominal Abcess, W. H. Fitch.............................. 225
Reviews and Book Notices..... 197, 287, 439, 523, 666
Reynolds, Henry J., Treatment of Eczema......................... 240
Rohe, Prof. Geo. H............... 91
Rosecrans, H., Letter........... 284
Rumination among the Insane................................ 224

S.

Sanitary Spelling Schools, with some Incidental Remarks concerning Dirty Linen....... 187
Selections......103, 209, 324, 530, 671
Sepulture, Review................. 198
Sexual Impotence in the Male, Review............................. 670
Sims, J. Marion..................... 111
Skeer, J. D., Malignant Stricture of the Descending Colon 408
Society Reports, 74, 166, 250, 410, 497, 631
Spinal Meningocele................ 317
Spontaneous Rupture of the Abdominal Walls in Inguinal Hernia............................. 291
Spray, J. C., Construction of Hospitals for the Insane...... 390
Strangulated Hernia, Coffee in 81
Stroinski, O., Translation........ 291
Successful Injection of a Solution of Salt in Acute Anæmia 110
Successive Dropsies of the Amnion always Specific...... 103
Sugar as an Antiseptic........... 309
Syphilis of the Brain.............. 297

PAGE.

T.

Tibbetts, J. H., Secretary Pathological Society............ 409
Therapeutic Ataxia of Hysteria 102
Trichiasis, Treatment of by Electrolysis, J. Elliot Colburn.................................. 39
Tonsilitis, Acute...................... 209
Treatment of Chronic Urithritis and Blenorrhagic Cystitis.................................. 311
Treatment of Diphtheria with the Cyanide of Mercury...... 102
Treatment of Eczema, Reynolds.................................. 240
Treatment of Emphysema in Infancy; Original Translation, E. S. McKee.............. 199
Treatment of Polypi of the Ear with Alcohol...................... 310
Treatment of Trichiasis by Electrolysis. J. Elliot Colburn................................. 39
Treatment of Uræmia............ 298
Treatment of Warts with Magnesia.................................. 224
Treatise on Insanity in its Medical Relations, Review... 4[illegible]9
Tuberculosis of the Choroidea 303

W.

Warts, Treatment of............. 224
Washburne, W. H., Resection of the Elbow...................... 131
Washburne, W. H. Case of Recovery............................... 468
Webster, C. E., Pott's Disease.. 357
Woman with Four Mammary Glands............................. 529
Wood, Dr. H. C., Notice......... 179

X.

Xanthelasma........................... 294

Y.

Yandell, David W................. 183
Year-Book of Surgery, Review 523
Year-Book of Therapeutics, Review............................... 525

